# Development and validation of a nomogram for the early prediction of acute kidney injury in hospitalized COVID-19 patients

**DOI:** 10.3389/fpubh.2022.1047073

**Published:** 2022-11-24

**Authors:** Congjie Wang, Huiyuan Sun, Xinna Li, Daoxu Wu, Xiaoqing Chen, Shenchun Zou, Tingshu Jiang, Changjun Lv

**Affiliations:** ^1^Pulmonary and Critical Care Medicine, Yantai Yuhuangding Hospital, Yantai, Shandong, China; ^2^Department of Critical Care Medicine, Yantai Yuhuangding Hospital, Yantai, Shandong, China; ^3^Department of Pathology, Yantai Yuhuangding Hospital, Yantai, Shandong, China; ^4^Department of Nephrology, Yantai Yuhuangding Hospital, Yantai, Shandong, China; ^5^Department of Respiratory Medicine, Binzhou Medical University Hospital, Binzhou Medical University, Binzhou, China

**Keywords:** COVID-19, acute kidney injury, nomogram, mortality, length of stay

## Abstract

**Introduction:**

Acute kidney injury (AKI) is a prevalent complication of coronavirus disease 2019 (COVID-19) and is closely linked with a poorer prognosis. The aim of this study was to develop and validate an easy-to-use and accurate early prediction model for AKI in hospitalized COVID-19 patients.

**Methods:**

Data from 480 COVID-19-positive patients (336 in the training set and 144 in the validation set) were obtained from the public database of the Cancer Imaging Archive (TCIA). The least absolute shrinkage and selection operator (LASSO) regression method and multivariate logistic regression were used to screen potential predictive factors to construct the prediction nomogram. Receiver operating curves (ROC), calibration curves, as well as decision curve analysis (DCA) were adopted to assess the effectiveness of the nomogram. The prognostic value of the nomogram was also examined.

**Results:**

A predictive nomogram for AKI was developed based on arterial oxygen saturation, procalcitonin, C-reactive protein, glomerular filtration rate, and the history of coronary artery disease. In the training set, the nomogram produced an AUC of 0.831 (95% confidence interval [CI]: 0.774–0.889) with a sensitivity of 85.2% and a specificity of 69.9%. In the validation set, the nomogram produced an AUC of 0.810 (95% CI: 0.737–0.871) with a sensitivity of 77.4% and a specificity of 78.8%. The calibration curve shows that the nomogram exhibited excellent calibration and fit in both the training and validation sets. DCA suggested that the nomogram has promising clinical effectiveness. In addition, the median length of stay (m-LS) for patients in the high-risk group for AKI (risk score ≥ 0.122) was 14.0 days (95% CI: 11.3–16.7 days), which was significantly longer than 8.0 days (95% CI: 7.1–8.9 days) for patients in the low-risk group (risk score <0.122) (hazard ratio (HR): 1.98, 95% CI: 1.55–2.53, *p* < 0.001). Moreover, the mortality rate was also significantly higher in the high-risk group than that in the low-risk group (20.6 vs. 2.9%, odd ratio (OR):8.61, 95%CI: 3.45–21.52).

**Conclusions:**

The newly constructed nomogram model could accurately identify potential COVID-19 patients who may experience AKI during hospitalization at the very beginning of their admission and may be useful for informing clinical prognosis.

## Introduction

In December 2019, Wuhan, China, reported the emergence of new coronavirus-associated pneumonia brought on by the novel SARS-CoV-2 infection (1, 2). On February 12, 2020, the World Health Organization (WHO) formally identified it as coronavirus disease 2019 (COVID-19), and on March 11, 2020, it was deemed a global pandemic. As of March 30, 2022, 227 countries and territories have been affected worldwide, with cumulatively more than 485 million cases confirmed, with over 6 million deaths (3). The main clinical feature of COVID-19 is acute respiratory symptoms (1, 2, 4, 5). Depending on the severity of the disease, patients can present with mild infections with no symptoms; moderate infections with symptoms such as fever, cough, and dyspnea; or even severe infections with acute respiratory distress syndrome (ARDS) (6, 7).

Although COVID-19 is a respiratory illness, it often results in multisystem damage that further progresses to multiple organ failure (MODS) and even, in severe cases, to patient death (4–7). The kidney is an important target organ for COVID-19 infection, and viral invasion causes acute kidney injury (AKI) through direct attack, an inflammatory storm, and inflammatory cell infiltration (8–10). The global incidence of COVID-19 in combination with AKI ranges from 0. 5 to 80%, and the incidence of AKI in the intensive care unit (ICU) ranges from 6 to 80% (11). The incidence of AKI significantly increases after COVID-19 infection (10, 12, 13). Studies have revealed that, compared to those hospitalized for non-COVID-19 reasons, COVID-19-infected hospitalized patients have an increased prevalence of AKI (31.0 vs. 18.0%) (14). A meta-analysis of 13,137 patients showed that the incidence of AKI in patients with COVID-19 was 17% (11). While, two observational studies that included 6,477 and 5,216 patients, respectively, revealed that the incidence of AKI among hospitalized COVID-19 patients was as high as 32 and 37% (15, 16). AKI increased the frequency and risk of mechanical ventilation in COVID-19 patients and lengthened their hospital stays. In addition, close to half of AKI patients did not have full recovery of renal function to baseline on discharge (16). Moreover, the incidence of AKI is linked with hospital mortality in patients with COVID-19 infection and is an independent risk factor for poor prognosis in critically ill patients (10, 12, 13, 17). A study that included 3,099 adult patients in critical condition who had COVID-19 showed that 20.6% of patients had to undergo kidney replacement therapy (KRT) for severe AKI within 14 days of entry to the intensive care unit. On day 28, the overall mortality rate for these patients was 54.9%, and up to 63.3% by the time of the last follow-up (17 days). Even among patients who were eventually discharged with a cure, there were still 33.6% of them dependent on KRT at discharge, and more than 50% of these patients still relied on KRT for the following 2 months (18). An autopsy study of patients who died from COVID-19 revealed that AKI was observed in 93.9% of patients, and 62% of patients experienced acute tubular necrosis of a different degree (14). Therefore, early clinical identification of patients who are at high risk for AKI could optimize the allocation of medical resources and enhance intervention management, thereby improving prognosis and reducing mortality.

Hence, we aimed to apply a new method to establish and validate a simple-to-use and effective early prediction model for AKI in hospitalized COVID-19 patients based on clinical characteristics, past medical history, clinical symptoms, signs, and key laboratory biochemical indicators. The model could help clinicians to screen patients with COVID-19 for the risk of AKI to identify and intervene in the early development of AKI. Furthermore, we investigated the prognostic differences between patients with high- and low-risk AKI based on predictive models.

## Methods

### Data collection and study design

Data from 480 COVID-19-positive patients were obtained from the public database of the Cancer Imaging Archive (TCIA) (collection of COVID-19-NY-SBU). This collection of patients was acquired at Stony Brook University with associated clinical data. AKI was defined as: (1) An increase in serum creatinine of 0.3 mg/dL within 48 h; (2) A rise in serum creatinine that is known or suspected to have happened within the previous 7 days, increasing it to 1.5 times baseline (or 50% above baseline); (3) Urine volume <0.5 ml/kg/h for 6 h. The inclusion criteria are as follows: (1) Age ≥18 years (weight ≥ 35Kg); (2) Laboratory-confirmed COVID-19 [positive polymerase chain reaction (PCR)]; (3) Expected hospital stay longer than 48 h; (4) With complete clinical information and laboratory test results. The exclusion criteria are as follows: (1) The Previous history of confirmed COVID-19. (2) The patient has received prophylactic treatment for COVID-19 within the last 30 days. (3) Patients with underlying renal disease such as chronic renal failure or post-transplantation or those on continuous renal replacement therapy, hemodialysis, or peritoneal dialysis. (4) Other diseases that may affect kidney function, such as tuberculous kidney disease, immune nephritis, and kidney tumors. (5) Presence of other serious diseases that damage life expectancy, such as acute myocardial infarction, cerebral hemorrhage, and pulmonary embolism. (6) Pregnancy and breastfeeding. The following information was collected for each patient: (1) general clinical characteristics of age, gender, and smoking history; (2) past medical history of hypertension, coronary artery disease (CAD), chronic obstructive pulmonary disease (COPD), and other lung diseases; (3) home medication history of an angiotensin-converting enzyme inhibitor (ACEI), angiotensin receptor blocker (ARB), antibiotics, and non-steroidal anti-inflammatory drugs (NSAID); (4) clinical symptoms of fever, cough, dyspnea, vomiting, diarrhea, and abdominal pain; (5) signs of oral temperature, arterial oxygen saturation (SaO2), respiratory rate, heart rate, systolic blood pressure, and mean blood pressure; (6) laboratory indicators of leukocyte count, neutrophils count, lymphocytes count, aspartate aminotransferase (AST), alanine aminotransferase (ALT), procalcitonin (PCT), C-reactive protein (CRP), sodium, potassium, chloride, lactate, blood urea nitrogen (BUN), serum creatinine (SCR), glomerular filtration rate (GFR), and glucose. All cohort patients were randomly divided into two sets at a ratio of 7:3: the training set was used to construct the prediction model, and the validation set was used to evaluate the performance of the model. The study was approved by the Ethics Committee of Yantai Yuhuangding hospital and conducted in accordance with the ethical principles of the Declaration of Helsinki. As all of the data in this work were retrieved from free online databases, informed consent was waived.

### Element selection and construction of the nomogram

The least absolute shrinkage and selection operator (LASSO) regression method was utilized in the training set in order to eliminate potentially predictive elements for AKI. LASSO regression analysis was performed to gain refinement of the model by constructing a penalization function, and applicable to regression analysis of high-dimensional data with multiple covariates. In the process of parameter selection, the LASSO regression automatically shrinks the regression coefficients of 41 parameters using the penalty parameter lambda (λ). The larger the value of lambda (λ), the more the coefficients of the parameters shrink to zero. Consequently, some parameters are eliminated due to the narrowing of their coefficients to near zero, while the remaining parameters are ultimately selected. Cross-validation was adopted to validate the adjustment parameter lambda (λ) appropriateness for the LASSO regression. The lambda (λ) parameter with minimum criteria of mean-squared error was selected to screen the potential predictive elements. Factors screened in LASSO regression were subsequently analyzed in a multivariate logistic regression model to identify significant predictors of AKI in hospitalized COVID-19 patients. To avoid overfitting, elements in the multivariate logistic regression model with a *p*-value < 0.1 were used to construct the prediction nomogram.

### Validation of the nomogram

Boost bootstrapping validation (1,000 bootstrap resamples) was used to evaluate the predictive effectiveness of the nomogram model in both the training- and validation sets. The performance metrics include the receiver operating curves (ROC), calibration curves, as well as decision curve analysis (DCA). The ROC and corresponding area under the curve (AUC) were utilized to quantify the discriminatory ability of the AKI nomogram. The AUC can be calculated by the integration of the area under the line segments, it ranges from 0.5 to 1.0, with 0.5 indicating a random and 1.0 indicating a perfectly differentiated. To evaluate the nomogram's identification and calibration, calibration curves were constructed. The Hosmer–Lemeshow test was performed to estimate the goodness-of-fit of the nomogram. To assess the nomogram model's clinical applicability and overall benefit, decision curve analysis (DCA) was utilized. DCA is an efficacious approach to the evaluation of the clinical benefits of alternative models, and when employed in nomograms, it can quantify the net benefits by performing at variable threshold probabilities. The DCA plotted the all-patient treatment scenario and the no-patient treatment scenario as two reference curves. The net benefit was calculated by deducting false-positive patients from true-positive patients, weighted by the potential damage of going untreated vs. the detrimental effects of going needless treatment. When the decision curve reveals that the nomogram is of greater benefit than the all-patient treatment scenario and the no-patient treatment scenario, it would indicate that the nomogram is clinically valid.

### Nomogram-based risk-group stratification

Based on the nomogram, risk scores for AKI were calculated for each patient, and patients were then divided into high- and low-risk cohorts based on the optimal cutoff value determined by the Youden index from the ROC analysis of the training set. Differences in length of stay and last status (discharged or deceased) of patients in the high- and low-risk cohorts were compared in both training and validation sets, respectively.

### Statistical analysis

The categorical variables were compared using Pearson's chi-square test and presented as percentages (%). For continuous variables, the Shapiro-Wilk test was used to test for normality, if the variables were normally distributed, the mean (standard deviation) was used for statistical description and the *t*-test was used for comparison between groups; otherwise, the medians [interquartile ranges (IQRs)] was used for statistical description and the Mann–Whitney *U*-test was used for comparison between groups. The median length of stay (m-LS) was calculated using the Kaplan-Meier approach, and the log-rank test was utilized to compare differences between high-risk and low-risk groups. The Cox proportional hazards model was used to determine the hazard ratio (HR) and its associated 95% confidence interval (CI). The mortality rate was compared by Fisher's exact or chi-squared tests, and odds ratios (ORs) with 95% CIs were calculated by logistic regression models. Statistical analysis was performed with the SPSS program (V22.0, Inc., Chicago, IL, USA) and R project (version 4.1.3, “glmnet” packages for LASSO logistic regression analysis, “forestplot” packages for plot forest, “hmisc” package for plot nomogram, “calibration curves” package for plot calibration curves, “pROC” package for plot ROC curves and calculate AUCs, and “stdca” package for DCA). A *p*-value < 0.05 (two-sided) was considered statistically significant.

## Results

### Characteristics of patients

**Figure 1:**
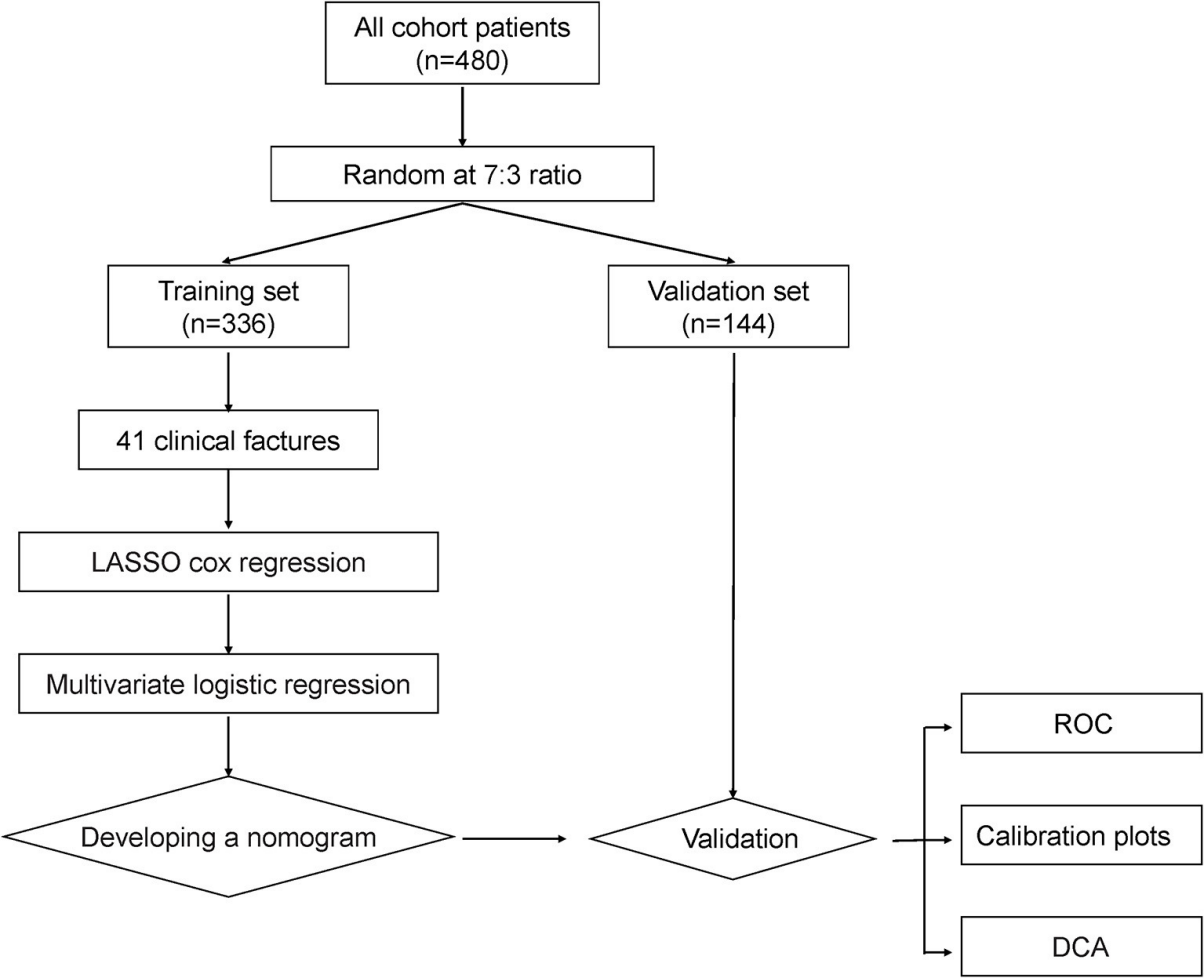
The flowchart of the study procedure. Abbreviations: LASSO, least absolute shrinkage and selection operator; ROC, receiver operating characteristic; DCA, decision curve analysis.

In total, 480 patients with COVID-19-positive were included in this study; 336 were randomized into the training set, while the remaining 144 were randomized into the validation set. The flowchart of the study procedure was present in Figure 1. The baseline characteristics of the two sets of patients were essentially balanced. The incidence of AKI was 17.7% (85 of 480) in the overall population, 16.1% (54 of 336) in the training set, and 21.5% (31 of 144) in the validation set, respectively (Table 1). Supplementary Table 1 compares the baseline characteristics of patients with AKI and those without AKI. For the whole cohort, there were slightly more patients aged <60 years (256, 53.3%) than those aged ≥60 years (224, 46.7%). There were 300 male patients (62.5%), which was more than the number of female patients (180, 37.5%). The majority of patients presented with infectious and respiratory symptoms, of which 395 (82.3%) patients presented with fever, 402 (83.8%) with cough, and 370 (77.1%) with dyspnea, while there was a relatively low frequency of gastrointestinal symptoms. Of those 480 patients, 21 patients received kidney replacement therapy, and 14 received kidney transplants. For patients receiving kidney replacement therapy, the mortality rate was 52.4% (11 of 21) and the mean length of stay was 39 days (only for discharged patients). For patients receiving received a kidney transplant, the mortality rate was 0.00% with a mean length of stay was 14.6 days. The baseline information for patients, including laboratory indicators, is detailed in Table 1.

**Table 1:** Baseline characteristics of patients in training set, validation set and all populations.

**Characteristic**	**All patients (*n* = 480)**	**Training set (*n* = 336)**	**Validation set (*n* = 144)**	**P value**
Age				
≥60	224 (46.7%)	153 (45.5%)	71 (49.3%)	0.448
<60	256 (53.3%)	183 (54.5)	73 (50.7%)	
Gender				
Male	300 (62.5%)	204 (60.7%)	96 (66.7%)	0.217
Female	180 (37.5%)	132 (39.3%)	48 (33.3%)	
Smoking				
Yes	123 (25.6%)	82 (24.4%)	41 (28.5%)	0.350
No	357 (74.4%)	254 (75.6%)	103 (71.5%)	
Hypertension				
Yes	236 (49.2%)	173 (51.5%)	63 (43.8%)	0.120
No	244 (50.8%)	163 (48.5%)	81 (56.2%)	
Diabetes				
Yes	130 (27.1%)	87 (25.9%)	43 (29.9%)	0.370
No	350 (72.9%)	249 (74.1%)	101 (70.1%)	
CAD				
Yes	58 (12.1%)	40 (11.9%)	18 (12.5%)	0.855
No	422 (87.9%)	296 (88.1%)	126 (87.5%)	
COPD				
Yes	18 (3.8%)	11 (3.3%)	7 (4.9%)	0.402
No	462 (96.2%)	325 (96.7%)	137 (95.1%)	
OLD				
Yes	72 (15.0%)	44 (13.1%)	28 (19.4%)	0.074
No	408 (85.0%)	292 (86.9%)	116 (80.6%)	
Malignancies				
Yes	37 (7.7%)	29 (8.6%)	8 (5.6%)	0.247
No	443 (92.3%)	307 (91.4%)	136 (94.4%)	
ACEI				
Yes	72 (15.0%)	47 (14.0%)	25 (17.4%)	0.343
No	408 (85.0%)	289 (86.0%)	119 (82.6%)	
ARB				
Yes	72 (15.0%)	53 (15.8%)	19 (13.2%)	0.468
No	408 (85.0%)	283 (84.2%)	125 (86.8%)	
Antibiotic				
Yes	139 (29.0%)	92 (27.4%)	47 (32.6%)	0.244
No	341 (71.0%)	244 (72.6%)	97 (67.6%)	
NSAID				
Yes	39 (8.1%)	305 (90.8%)	136 (94.4%)	0.177
No	441 (91.9%)	31 (9.2%)	8 (5.6%)	
Fever				
Yes	395 (82.3%)	277 (82.4%)	118 (81.9%)	0.896
No	85 (17.7%)	59 (17.6%)	26 (18.1%)	
Cough				
Yes	402 (83.8%)	281 (83.6%)	121 (84.0%)	0.914
No	78 (16.3%)	55 (16.4%)	23 (16.0%)	
Dyspnea				
Yes	370 (77.1%)	252 (75.0%)	118 (81.9%)	0.095
No	110 (22.9%)	84 (25.0%)	26 (18.1%)	
Vomiting				
Yes	89 (18.5%)	64 (19.0%)	25 (17.4%)	0.663
No	391 (81.5%)	272 (81.0%)	119 (82.6%)	
Diarrhea				
Yes	191 (39.8%)	141 (42.0%)	50 (34.7%)	0.137
No	289 (60.2%)	195 (58.0%)	94 (65.3%)	
Abdominal pain				
Yes	65 (13.5%)	49 (14.6%)	16 (11.1%)	0.308
No	415 (86.5%)	287 (85.4%)	128 (88.9%)	
T				
(°C)	37.50 (37.00, 38.30)	37.60 (37.10, 38.40)	37.40 (37.00, 38.10)	0.157
SaO2				
	94.00 (91.00, 96.00)	94.00 (91.00, 96.00)	93.00 (91.00, 96.00)	0.209
PR				
(#/min)	20.00 (18.00, 24.00)	20.00 (18.00, 24.00)	20.00 (18.00, 25.00)	0.811
HR				
(#/min)	100.00 (88.00, 112.00)	100.00 (88.00, 113.00)	99.00 (88.00, 111.00)	0.484
SBP				
(mmHg)	125.00 (113.00, 142.00)	125.00 (112.00, 142.00)	125.00 (113.00, 141.00)	0.944
MAP				
(mmHg)	91.00 (84.00, 99.00)	91.00 (82.00, 98.00)	91.00 (85.00, 99.00)	0.469
Leukocytes				
(#/volume)	6.83 (5.22, 8.81)	6.69 (5.21, 8.72)	7.44 (5.50, 9.10)	0.108
Neutrophils				
(#/volume)	5.27 (3.84, 7.14)	5.12 (3.76, 6.98)	5.69 (4.08, 7.33)	0.071
Lymphocytes				
(#/volume)	0.93 (0.67, 1.25)	0.93 (0.66, 1.25)	0.94 (0.67, 1.25)	0.985
AST				
(U/volume)	42.00 (29.00, 64.00)	42.00 (30.00, 63.00)	42.00 (28.00, 66.00)	0.715
ALT				
(U/volume)	33.00 (22.00, 55.00)	33.00 (22.00, 55.00)	33.00 (21.00, 55.00)	0.731
PCT				
(moles/volume)	0.16 (0.10, 0.29)	0.16 (0.10, 0.30)	0.16 (0.09, 0.28)	0.768
CRP				
(moles/volume)	8.60 (3.80, 14.20)	8.60 (4.00, 14.40)	8.70 (3.60, 14.00)	0.663
Sodium				
(moles/volume)	136.00 (133.00, 138.00)	136.00 (133.00, 138.00)	136.00 (133.00, 139.00)	0.312
Potassium				
(moles/volume)	4.10 (3.80, 4.40)	4.10 (3.80, 4.40)	4.20 (3.80, 4.50)	0.164
Chloride				
(moles/volume)	97.00 (94.00, 99.00)	97.00 (94.00, 99.00)	98.00 (94.00, 99.00)	0.403
Lactate				
(moles/volume)	1.40 (1.10, 1.80)	1.40 (1.10, 1.80)	1.50 (1.10, 1.80)	0.514
Bicarbonate				
(moles/volume)	24.00 (22.00, 25.00)	24.00 (22.00, 25.00)	24.00 (22.00, 26.00)	0.183
BUN				
(mass/volume)	13.00 (10.00, 20.00)	13.00 (9.00, 20.00)	14.00 (11.00, 21.00)	0.368
SCR				
(mass/volume)	0.91 (0.72, 1.16)	0.89 (0.70, 1.13)	0.98 (0.77, 1.27)	0.043
GFR				
(ml/min)	0.91 (0.72, 1.16)	0.89 (0.70, 1.13)	0.98 (0.77, 1.27)	0.213
Glucose				
(mass/volume)	120.00 (107.00, 151.00)	120.00 (106.00, 147.00)	121.00 (110.00, 159.00)	0.181
AKI	17.7%	16.1%	21.5%	0.151

Abbreviations: CAD, coronary artery disease; COPD, chronic obstructive pulmonary disease; OLD, other lung diseases including asthma; ACEI, angiotensin converting enzyme inhibitor; ARB, Angiotensin receptor blocker; NSAID, non-steroidal anti-inflammatory drug; T, temperature; SaO2, artery oxygen saturation; PR, respiration rate; HR, heart rate; SBP, systolic blood pressure; MAP, mean blood pressure; AST, aspartate aminotransferase; ALT, alanine aminotransferase; PCT, procalcitonin; CRP, C-reactive protein; BUN, blood urea nitrogen; SCR, serum creatinine; GFR, glomerular filtration rate.

### Feature selection

**Figure 2:**
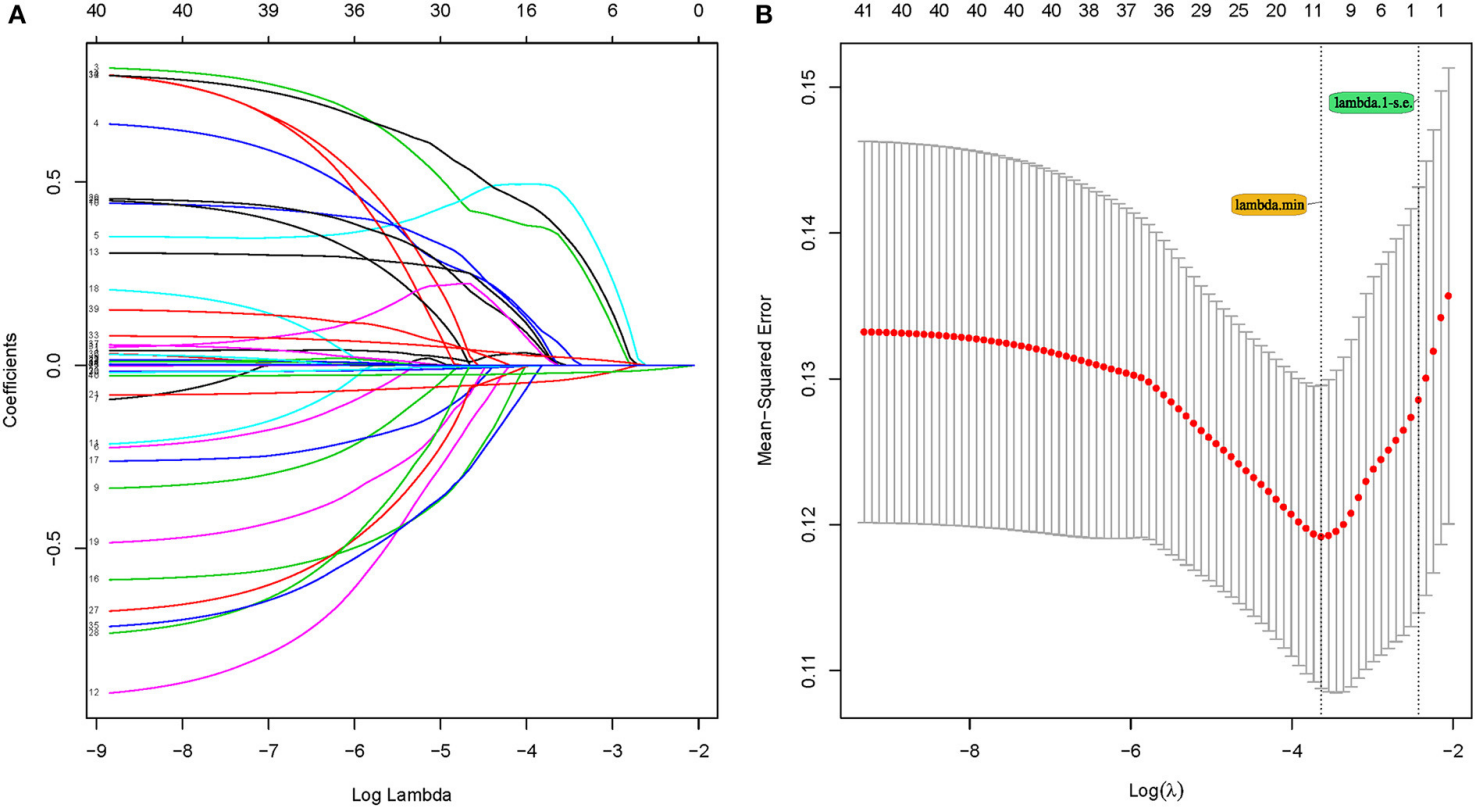
Feature selection using the least absolute shrinkage and selection operator (LASSO) Cox regression model. **(A)** LASSO coefficient profiles of the 41 features. **(A)** coefficient profile plot was produced against the log (λ) sequence. **(B)** Selection of tuning parameter (λ) in the LASSO regression using 10-fold cross-validation *via* minimum criteria. At the optimal values log (λ), where features are selected, two dotted vertical lines were drawn at the optimal scores by minimum criteria and 1-s.e. criteria.

Feature selection was performed in the training set, and 41 parameters were included in the LASSO logistic regression analysis for predictor screening. The coefficient profile plot was produced against the log (λ) sequence (Figure 2A). Minimum criteria are used to select the tuning parameter (λ) for the LASSO regression utilizing 10-fold cross-validation. The results show that the optimal value of tuning parameter λ in the LASSO logistic regression was 0.026 when the mean-squared error reached its minimum value. Ten parameters with non-zero coefficients were screened: hypertension history, CAD, diabetes, SaO2, ALT, lactate, PCT, CRP, SCR, and GFR (Figure 2).

### Construction of the nomogram and performance examination

**Figure 3:**
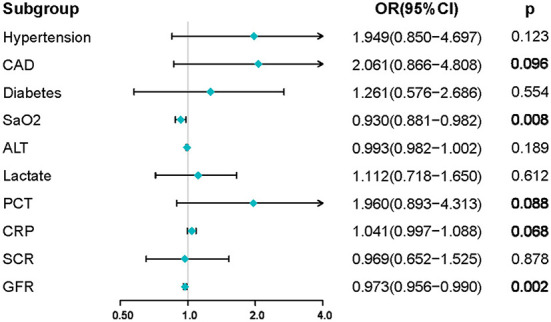
Results of multivariate logistic regression analysis in the training set. Factors with *p*-values < 0.1 were screened for constructing the nomogram model. Abbreviations: CAD, coronary artery disease; SaO2, artery oxygen saturation; ALT, alanine aminotransferase; PCT, procalcitonin; CRP, C-reactive protein; GFR, SCR, serum creatinine; GFR, glomerular filtration rate.

**Figure 4:**
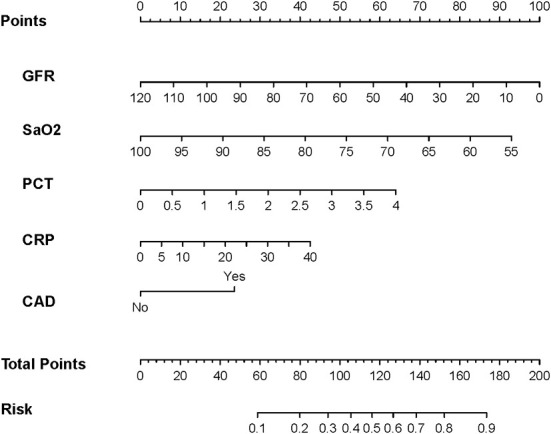
The nomogram was developed in the training set. It included five factors: glomerular filtration rate (GFR), artery oxygen saturation (SaO2), procalcitonin (PCT), C-reactive protein (CRP), and history of coronary artery disease (CAD). The nomogram plot provides a visual way to predict the risk of AKI for COVID-19 patients.

**Figure 5:**
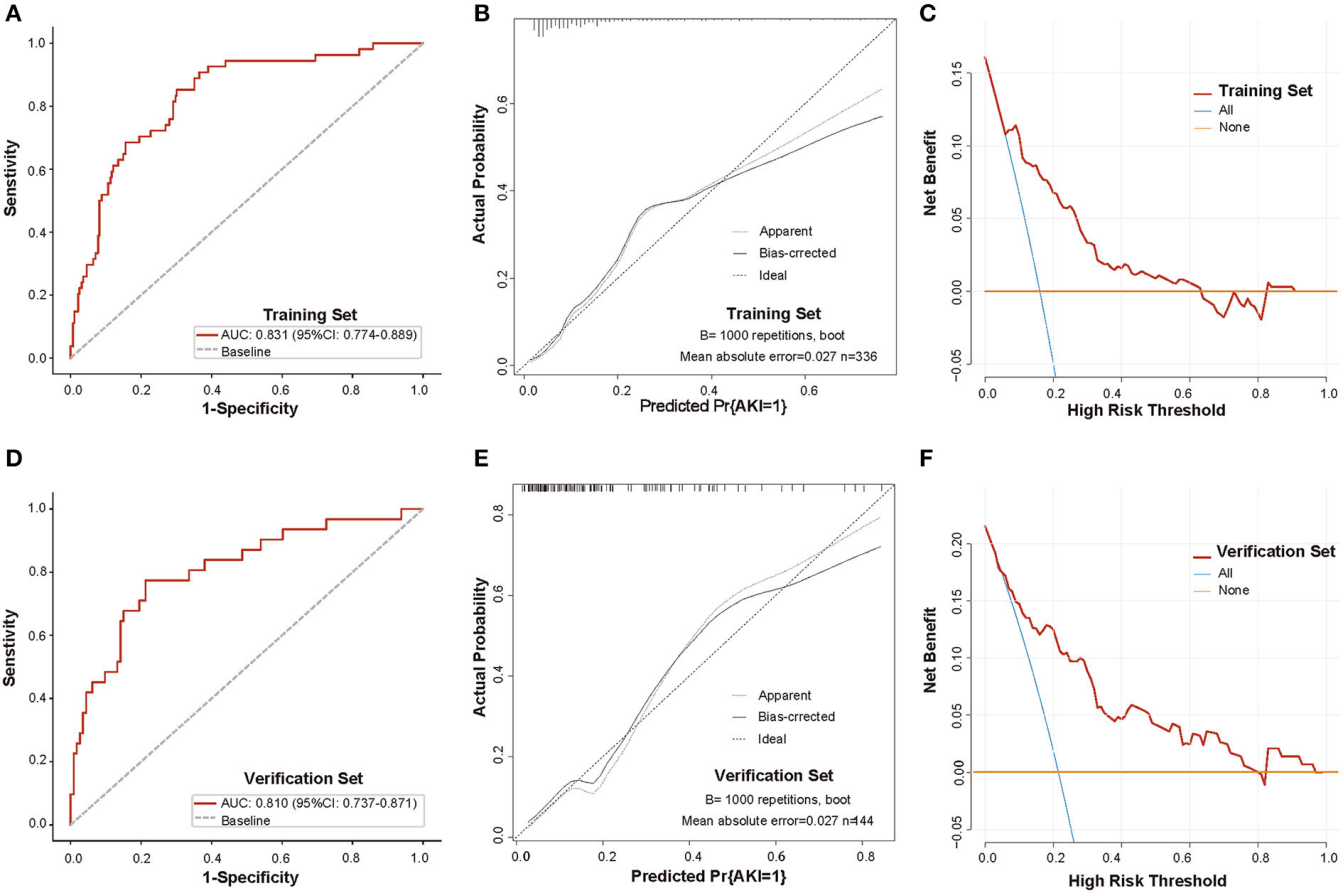
Validation of the discrimination power of the nomogram in the training and validation sets. **(A,D)** ROC curve analysis of the nomogram in the training and validation sets (AUC, 0.831 and 0.810, respectively); **(B,E)** Calibration plot of the nomogram in the training and validation sets, The black dashed diagonal line indicates the perfect prediction of the ideal model. The solid black line represents the performance of the nomogram, and the closer the fit to the diagonal line, the more accurate the prediction. The gray dashed line represents the performance of the model trained after bootstrapping validation (1,000 bootstrap resamples), which corrects the overfitting situation; **(C,F)** DCA analysis of the nomogram in the training and validation sets. The y-axis represents the net benefit, the x-axis represents the threshold probability. The red line represents the nomogram, and the blue and orange lines represent the all-patient treatment scenario and the no-patient treatment scenario, respectively. Abbreviations: ROC, receiver operating characteristic; AUC, area under the curve; DCA, decision curve analysis.

The parameters screened in the LASSO regression were utilized for the multivariate logistic regression model analysis, and the results demonstrated that only SaO2 and GFR were independent predictors of the occurrence of AKI (SaO2, OR:0.930, 95% CI: 0.881–0.982, *P* = 0.008; GFR, OR:0.973, 95% CI: 0.956–0.990, *P* = 0.002) (Figure 3). However, to avoid overfitting of the nomogram model, parameters with *p* < 0.1 were selected for model construction. Finally, a predictive nomogram model for the occurrence of AKI in hospitalized COVID-19 patients based on CAD, SaO2, PCT, CRP, and GFR was constructed (Figure 4, Supplementary Table 2). Based on the nomogram, the point scale scores for these five independent variables could be calculated for each patient, and their sum was the total point value. The ROC curve showed that the nomogram had favorable discrimination for AKI, with an AUC of 0.831 (95% CI: 0.774–0.889), a sensitivity of 85.2%, and a specificity of 69.9% (Figure 5A), which was significantly better than those of SCR and BUN (Supplementary Figure 1A). The calibration curves visually revealed favorable accordance between the prediction of the nomogram and the actual observations (Figure 5B). The Hosmer–Lemeshow test demonstrated a nice goodness-of-fit of the nomogram, with no significant differences observed (*p* = 0.247). DCA showed that the nomogram had a nice overall net benefit in the threshold probability range of 16–63%, and was superior to those of SCR and BUN (Supplementary Figure 1C), indicating that the model has promising clinical effectiveness (Figure 5C).

### Validation of the nomogram

Next, we evaluated the effectiveness of the model in the validation set. Consistent with the results of the training set, the nomogram yielded a favorable AUC of the ROC curve of 0.810 (95% CI: 0.737–0.871), with a sensitivity of 77.4% and specificity of 78.8% (Figure 5D), better than those of SCR and BUN (Supplementary Figure 1B). The calibration curve and Hosmer–Lemeshow test suggested that the nomogram had good calibration and fit in the validation set (*p* = 0.247) (Figure 5E). Moreover, DCA visually revealed that the nomogram had an overall net benefit within a wider threshold probability in the validation set (Figure 5F, Supplementary Figure 1D). These results suggest that the nomogram functions well and has excellent predictive power for the validation set.

### Nomogram-based risk stratification

**Figure 6:**
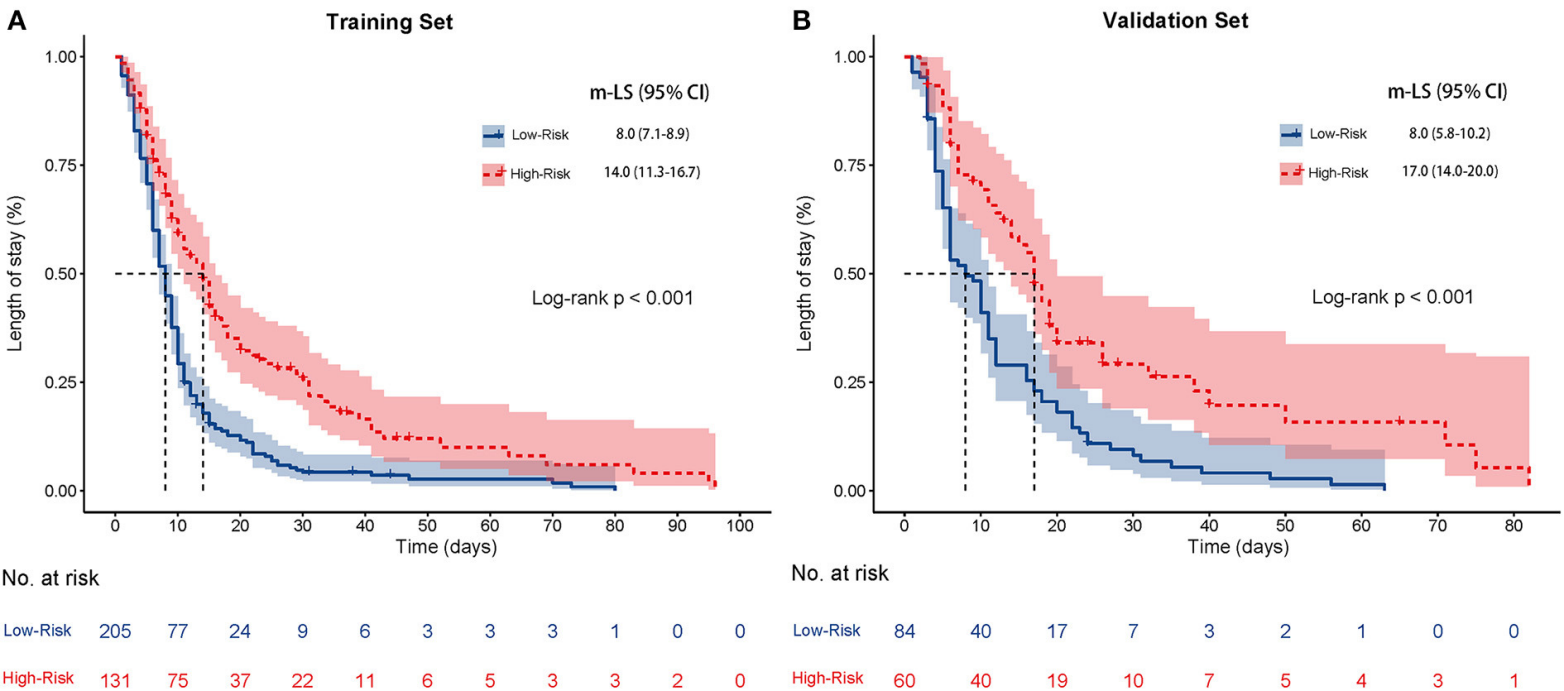
Kaplan-Meier curves for the length of stay of high-risk patients and low-risk patients based on the optimal segmentation threshold obtained from the ROC analysis in the training set **(A)** and the validation set **(B)**. Abbreviations: m-LS: median length of stay.

Based on the nomogram constructed in the training set, the probability of occurrence of AKI was calculated for each patient. Using the optimal cutoff value of 0.122 obtained from the ROC analysis of the training set, the two sets of patients were subsequently classified into high- and low-risk groups. In the training set, there were 205 patients in the low-risk group and 131 patients in the high-risk group. The median length of stay (m-LS) for patients in the high-risk group was 14.0 days (95% CI: 11.3–16.7 days), which was significantly longer than 8.0 days (95% CI: 7.1–8.9 days) for patients in the low-risk group, which was (HR:1.98, 95%CI: 1.55–2.53, *p* < 0.001) (Figure 6A). Similarly, in the validation set, we also observed a significantly longer length of stay in the high-risk group than in the low-risk group (m-LS: 17.0 days vs. 8.0 days, HR: 2.10, 95% CI: 1.45–3.05, *p* < 0.001) (Figure 6B). In addition, we found that the mortality rate was higher in the high-risk group. In the training set, there were 27 patients in the high-risk group having a last status of death, with a mortality of 20.6%, compared to 2 (2.9%) in the low-risk group in the training set (OR: 8.61, 95% CI: 3.45–21.52, *p* < 0.001). The results from the validation set corroborated this finding, where the last status was deceased in 14 (23.3%) and 2 (2.4%) patients in each of the high- and low-risk groups, respectively, with statistically significant differences (OR: 12.48, 95% CI: 2.72–57.33, *p* = 0.001). These results indicate that the nomogram model can be applied to predict the prognosis of hospitalized COVID-19 patients (Table 2).

**Table 2:** Comparisons of last status and length of stay of patients on Nomogram-based risk stratification in training and validation set.

**Characteristics**	**No**.	**Last status**					**Length of stay (days)**		
		**Deceased**	**Discharged**	**Mortality**	**OR (95%CI)**	***P***	**m-LS (95%CI)**	**HR (95%CI)**	***P***
**Training set**									
High-risk	131	27	104	20.6%	8.61 (3.45–21.52)	**<**0.001	14.0 (11.3–16.7)	1.98 (1.55–2.53)	**<**0.001
Low–risk	205	6	199	2.9%			8.0 (7.1–8.9)		
**Validation Set**									
High–risk	60	14	46	23.3	12.48 (2.72–57.33)	0.001	17.0 (14.0–20.0)	2.10 (1.45–3.05)	**<**0.001
Low–risk	84	2	82	2.4%			8.0 (5.8–10.2)		

## Discussion

In this study, we developed a predictive nomogram model for acute kidney injury in hospitalized COVID-19 patients based on SaO2, PCT, CRP, GFR, and the history of CAD. It is an easy-to-use, well-performing nomogram model with promising discrimination, predictive accuracy, and clinical practical utility. In addition, the risk score based on the nomogram was related to the length of stay and the last status of the patient. This is the first nomogram model to predict AKI in COVID-19 inpatients at an early stage. It is conducive to the early identification of high-risk patients with AKI to provide early intervention and treatment, and it can effectively and reasonably optimize the allocation and utilization of hospital beds as well as medical resources, thereby alleviating the shortage of medical resources and improving patient prognosis.

Acute kidney injury is a group of clinical syndromes characterized by a rapid decline (hours to days) in kidney function (19) and is a common complication in patients hospitalized with COVID-19 (8–10, 12, 13, 17). Patients may present with urinary abnormalities, for example, proteinuria and hematuria, elevated blood creatinine and urea nitrogen, and even positivity for SARS-CoV-2 in urine tests (1, 2, 10, 13). According to previous studies, ~40% of patients with COVID-19 had proteinuria on admission, ~10% had elevated blood creatinine during the course of the disease, ~21% had elevated blood urea nitrogen, ~43% had persistently elevated blood urea nitrogen, ~63% had proteinuria, and ~26.9% had hematuria (20, 21). The incidence of AKI varied by medical center, race, statistical size of the sample, and severity of disease in the included population. For example, According to a study of 138 Wuhan residents who were diagnosed with COVID-19, the incidence of AKI was 3.6%, while the incidence of AKI in critically ill patients was 8.3% (2). Of 1,099 patients with COVID-19, Guan et al. (4) reported an incidence of AKI of 0.5%, with 5 of 17 (2.9%) critically ill patients developing AKI. In addition, another Chinese study enrolled 710 patients with COVID-19, 52 of whom were critically ill adults, with an AKI rate of 29% (21). An Italian study showed a 15% incidence of AKI in COVID-19 patients (22), and the results of another study of 5,700 patients with COVID-19 in New York reported an AKI incidence reaching as high as 22.2% (23). Altogether, the incidence of AKI in patients with COVID-19 ranges from 0.1 to 56.9%, while it reaches 77% in patients with severe COVID-19 (2, 4, 11, 21–23). Analyzing the previous evidence, the following information can be obtained. First, kidney injury is not uncommon in patients with COVID-19 (especially those with severe disease). Second, the incidence of AKI has been inconsistent, which is mainly related to the sample size and study population, and the incidence of AKI is higher in severe and critical COVID-19 patients. Most importantly, COVID-19 complicated with AKI is an independent risk factor for poor prognosis (11, 21–23). Among patients who died from COVID-19, the incidence of AKI was as high as 37.5%, which was significantly higher than the 15% of surviving cases (24). The mortality rate of COVID-19 patients complicated with AKI was reported to be 67% (95% CI: 39.8–86.2%), and the risk of death was 13 times that of patients without AKI (OR = 13.3, 95% CI: 6.1–29.2) (25). In addition, the severity of AKI is associated with patient prognosis, and patients with the late-stage disease have a significantly higher risk of death (2, 8, 10, 12). Therefore, early assessment of AKI risk is important to guide physicians to intervene early and prevent AKI, protect renal function, and avoid progression of the patient's condition.

A nomogram may provide a quantitative and pragmatic predictive tool for risk stratification of COVID-19 patients for the development of AKI during hospitalization. In this study, GFR, SaO2, PCT, CRP, and history of CAD were selected by LASSO and multivariate regression for the construction of the predictive nomogram model of AKI. These factors have been demonstrated to correlate with the development of AKI in previous studies. An early Chinese study including 701 patients with COVID-19 showed that patients were more likely to develop AKI if their admission baseline SCR levels were higher (11.9 vs. 4%) (21). Data from a retrospective study of 306 patients from Sweden demonstrated that decreased baseline renal function increases the risk of developing AKI during hospitalization in COVID-19 patients. The risk ratios for experiencing AKI in patients with an eGFR between 30 and 59 ml/min and an eGFR <30 ml/min were 2.94 (95% CI: 1.17–7.34) and 9.93 (95% CI: 2.32–42.5), respectively (26). An international multicenter study of 939 patients identified that poor respiratory function (lower oxygen saturation and PaO2/FiO2 ratio) was a risk factor for the development of AKI (27). In addition, studies have shown that inflammatory indicators, such as C-reactive protein (27) and procalcitonin (28); underlying diseases, such as hypertension, diabetes, and chronic kidney disease; as well as coronary artery disease, are correlated with the occurrence of AKI (29, 30). There are also predictive models (scores or biomarkers) for AKI in COVID-19 patients that have been reported in previous studies. Gustavo et al. (31) investigated the capability of urinary kidney stress biomarkers (UKSB), including neutrophil gelatinase-associated lipocalin (NGAL) and tissue inhibitor of metalloproteinases-2 (TIMP-2) multiplied by insulin-like growth factor binding protein 7 (IGFBP7), for the early detection of AKI in 51 critically ill COVID-19 patients. The results showed that the AUCs of the ROC for NGAL and TIMP-2 × IGFBP7 predicting the occurrence of AKI during the entire hospitalization of patients were 0.706 (95% CI: 0.559–0.854) and 0.682 (95% CI: 0.535–0.829), respectively, with corresponding sensitivities and specificities of 54.5, 76.9, 40.0, and 88.4%. Naomi et al. (32) reported the application of serum biomarkers (SB), including serum NGAL and serum creatinine, for the prediction of AKI in 52 COVID-19 patients, with AUCs of 0.81 and 0.87, respectively. A prediction model for AKI based on proteinuria and hematuria yielded an AUC of 0.64 (95% CI: 0.62–0.67) in a large cohort study containing 5,980 COVID-19 patients; moreover, the predictive capability of the model was improved when creatinine and the presence of CKD were incorporated (33). In addition, studies have validated the predictive value of other indicators, such as D-dimer and albumin/creatinine ratio, for AKI in hospitalized COVID-19 patients (34). However, these predictive models (biomarkers) have some disadvantages. First and foremost, their performance has not been validated, which leads to a lack of confidence in their reproducibility and utility. Second, they focused only on a particular type or class of indicators, which may present only a partial characterization of the patient's disease. Third, some models were constructed based on small sample sizes, such as the UKSB model and the SB model, which may not be representative of the whole cohort population. In contrast, in the present study, we screened indicators on the basis of seven dimensions of admission information for the construction of a predictive nomogram model of AKI during hospitalization based on a training set with 366 COVID-19 patients. Importantly, we verified the effectiveness of the nomogram in both training and validation cohorts and found that the nomogram displayed promising identification, goodness-of-fit, discriminative power, and clinical effectiveness.

We also investigated the predictive value of the model for patient prognosis. The mortality rate of patients in the high-risk group was higher than that of patients in the low-risk group; furthermore, the mortality rate of high-risk patients was greater than that of low-risk patients, with a hazard ratio of 1.98 for the training set and 2.10 for the validation set. This may be because high-risk patients are susceptible to the occurrence of AKI, while previous studies have demonstrated a higher mortality rate in COVID-19 patients with AKI (35). Regardless, the nomogram model could identify patients with a potentially poor prognosis at the beginning of their admission, which is helpful for the formulation of individualized treatment strategies and the arrangement of appropriate care and treatment at an early stage.

The study has several limitations. First, it is a public database-based study, and the results may have been influenced by confounding factors beyond our control. Second, although the model performed well in the validation set, we did not evaluate its performance in an independent external validation cohort. Third, the length of stay and last status of patients are influenced by other factors, especially treatment measures; therefore, relying on the model alone to predict prognosis is not sufficient. In addition, some other indicators, such as virus load or virus-related indicators, red blood cell count, and hemoglobin concentration, may have some correlation with the occurrence of AKI; however, we were unable to investigate these further due to the limitations of data availability, which may affect the reliability and stability of our conclusions. Moreover, due to the limitations of data availability, the difference in special treatment during hospitalization, such as hemodialysis technology, between high-risk and low-risk groups was not analyzed. Hence, the predictive capability for AKI and the prognostic value of the model needs to be verified in actual clinical practice.

## Conclusion

We constructed a nomogram for the early prediction of AKI in hospitalized COVID-19 patients. The model demonstrated favorable performance on the basis of the AUCs of ROC, calibration curves, and decision curve analysis. Furthermore, the nomogram exhibited promising predictive values for a prognosis for the length of stay and last status of patients. The nomogram model is helpful to reasonably and effectively optimize the allocation and utilization of medical resources at an early stage to provide appropriate care and intervention management for patients, thereby improving prognosis and reducing mortality.

## Data availability statement

The raw data supporting the conclusions of this article will be made available by the authors, without undue reservation.

## Ethics statement

The studies involving human participants were reviewed and approved by the Ethics Committee of Yantai Yuhuangding Hospital. Written informed consent for participation was not required for this study in accordance with the national legislation and the institutional requirements.

## Author contributions

Methodology: CW, XL, and DW. Software: XC, SZ, and CW. Formal analysis: CW, HS, and XC. Resources: TJ and XL. Data curation: HS and SZ. Writing—original draft preparation: CW and HS. Writing—review and editing, supervision, and project administration: CL and TJ. All authors contributed to the article and approved the submitted version.

## Conflict of interest

The authors declare that the research was conducted in the absence of any commercial or financial relationships that could be construed as a potential conflict of interest.

## Publisher's note

All claims expressed in this article are solely those of the authors and do not necessarily represent those of their affiliated organizations, or those of the publisher, the editors and the reviewers. Any product that may be evaluated in this article, or claim that may be made by its manufacturer, is not guaranteed or endorsed by the publisher.

## Supplementary material

Supplementary Figure 1 Comparison of AUCs between Nomogram with SCR and BUN in the training set **(A)** and validation set **(B)**. Comparison of DCAs between Nomogram with SCR and BUN in the training set **(C)** and validation set **(D)**. Abbreviations: ROC, receiver operating characteristic; AUC, area under the curve; DCA, decision curve analysis; SCR, serum creatinine; BUN, blood urea nitrogen.

Supplementary Table 1 Comparison of characteristics between patients with AKI and non-AKI.

Supplementary Table 2 The parameters of the final multifactor model for AKI.
