# Supplementary figures and images for: Development and validation of a nomogram for the early prediction of acute kidney injury in hospitalized COVID-19 patients
Source: Front Public Health. 2022 Nov 24;10:1047073. doi: 10.3389/fpubh.2022.1047073 (PMC9730715; doi:10.3389/fpubh.2022.1047073)

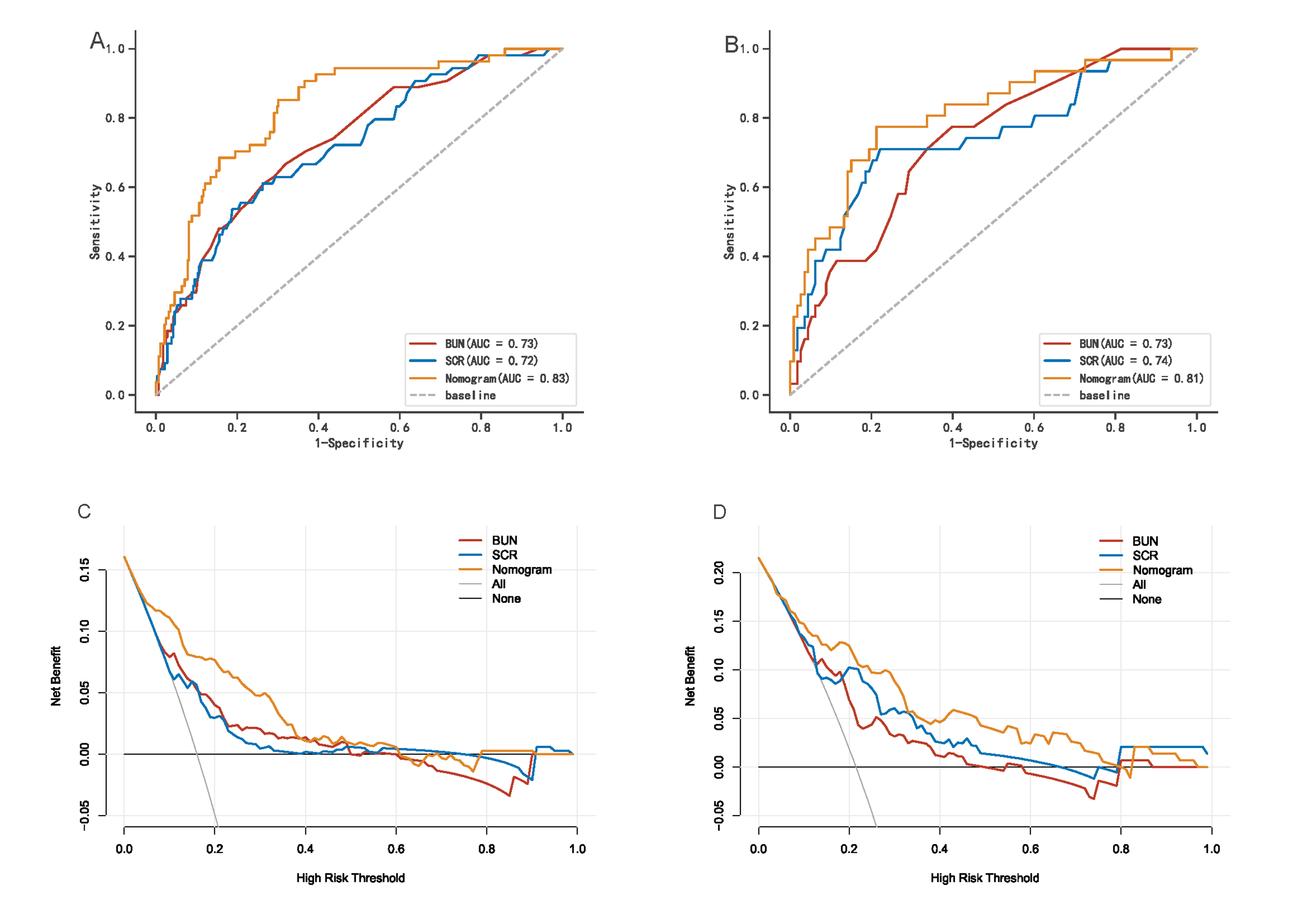

Supplement: Supplementary Figure 1 — Comparison of AUCs between Nomogram with SCR and BUN in the training set (A) and validation set (B). Comparison of DCAs between Nomogram with SCR and BUN in the training set (C) and validation set (D). Abbreviations: ROC, receiver operating characteristic; AUC, area under the curve; DCA, decision curve analysis; SCR, serum creatinine; BUN, blood urea nitrogen. [file Image_1.TIFF]
