# Supplementary material for: Development and validation of a nomogram for the early prediction of acute kidney injury in hospitalized COVID-19 patients
Source: Front Public Health. 2022 Nov 24;10:1047073. doi: 10.3389/fpubh.2022.1047073 (PMC9730715; doi:10.3389/fpubh.2022.1047073)
Supplement: Supplementary Table 1 — Comparison of characteristics between patients with AKI and non-AKI. [file Data_Sheet_1.DOCX]

Supplementary Table 1. Comparison of characteristics between patients with AKI and non-AKI.

| **Characteristic** | **AKI cohort**  **(n=85)** | **Non-AKI cohort (n=395)** | **P-value** |
| --- | --- | --- | --- |
| Age |  |  |  |
| ≥60 | 58(68.2%) | 166(42.0%) | <0.001 |
| <60 | 27(31.8) | 229(58.0%) |  |
| Gender |  |  |  |
| Male | 59(69.4%) | 241(61.0%) | 0.147 |
| Female | 26(20.6%) | 154(39.0%) |  |
| Smoking |  |  |  |
| Yes | 32(37.6%) | 91(23.0%) | 0.005 |
| No | 53(62.4%) | 304(77.0%) |  |
| Hypertension |  |  |  |
| Yes | 61(71.8%) | 175(44.3%) | 0.001 |
| No | 24(28.2%) | 220(55.7%) |  |
| Diabetes |  |  |  |
| Yes | 35(41.2%) | 95(24.1%) | 0.001 |
| No | 50(58.8%) | 300(75.9%) |  |
| CAD |  |  | <0.001 |
| Yes | 22(25.9%) | 36(9.1%) |  |
| No | 63(74.1%) | 359(90.9%) |  |
| COPD |  |  |  |
| Yes | 5(5.9%) | 13(3.3%) | 0.254 |
| No | 80(94.1%) | 382(96.7%) |  |
| OLD |  |  |  |
| Yes | 16(18.8%) | 56(14.2%) | 0.276 |
| No | 69(81.2%) | 339(85.8%) |  |
| Malignancies |  |  |  |
| Yes | 10(11.8%) | 27(6.8%) | 0.122 |
| No | 75(88.2%) | 368(93.2%) |  |
| ACEI |  |  |  |
| Yes | 16(18.8%) | 56(14.2%) | 0.276 |
| No | 69(81.2%) | 339(85.8%) |  |
| ARB |  |  |  |
| Yes | 20(23.5%) | 52(13.2%) | 0.015 |
| No | 65(76.5%) | 343(86.8%) |  |
| Antibiotic |  |  |  |
| Yes | 25(29.4%) | 114(28.9%) | 0.010 |
| No | 60(70.6%) | 281(71.1%) |  |
| NSAID |  |  |  |
| Yes | 3(3.5%) | 36(9.1%) | 0.087 |
| No | 82(96.5%) | 359(90.9%) |  |
| Fever |  |  |  |
| Yes | 67(78.8%) | 328(83.0%) | 0.356 |
| No | 18(21.2%) | 67(17.0%) |  |
| Cough |  |  |  |
| Yes | 67(78.8%) | 335(85.8%) | 0.175 |
| No | 18(21.2%) | 60(15.2%) |  |
| Dyspnea |  |  |  |
| Yes | 67(78.8%) | 303(76.7%) | 0.674 |
| No | 18(21.2%) | 92(23.3%) |  |
| Vomiting |  |  |  |
| Yes | 14(16.5%) | 75(19.0%) | 0.588 |
| No | 71(83.5%) | 320(81.0%) |  |
| Diarrhea |  |  |  |
| Yes | 29(34.1%) | 162(41.0%) | 0.239 |
| No | 56(65.9%) | 233(59.0%) |  |
| Abdominal pain |  |  |  |
| Yes | 11(13.0%) | 54(13.7%) | 0.858 |
| No | 74(87.0%) | 341(86.3%) |  |
| T |  |  |  |
| (℃) | 37.7[37.00,38.70] | 37.50[37.00,38.20] | 0.219 |
| SaO2 |  |  |  |
|  | 92.00[84.00,96.00] | 94.00[92.00,96.00] | <0.001 |
| PR |  |  |  |
| (#/min) | 22.00[18.00,28.00] | 20.00[18.00,24.00] | 0.015 |
| HR |  |  |  |
| (#/min) | 96.00[85.00,108.00] | 101.00[88.00,114.00] | 0.036 |
| SBP |  |  |  |
| (mmHg) | 128.00[113.00,142.00] | 125.00[112.00,142.00] | 0.703 |
| MAP |  |  |  |
| (mmHg) | 92.00[80.00,101.00] | 90.00[84.00,98.00] | 0.679 |
| Leukocytes |  |  |  |
| (#/volume) | 6.84[4.87,8.55] | 6.82[5.26,8.91] | 0.737 |
| Neutrophils |  |  |  |
| (#/volume) | 5.16[3.59,7.17] | 5.27[3.84,7.14] | 0.884 |
| Lymphocytes |  |  |  |
| (#/volume) | 0.81[0.59,1.06] | 0.96[0.70,1.30] | 0.002 |
| AST |  |  |  |
| (U/volume) | 45.00[34.00,65.00] | 41.00[29.00,64.00] | 0.174 |
| ALT |  |  |  |
| (U/volume) | 30.00[21.00,48.00] | 33.00[22.00,56.00] | 0.268 |
| PCT |  |  |  |
| (moles/volume) | 0.24[0.14,0.62] | 0.15[0.09,0.26] | <0.001 |
| CRP |  |  |  |
| (moles/volume) | 10.90[6.10,19.90] | 7.90[3.50,13.60] | <0.001 |
| Sodium |  |  |  |
| (moles/volume) | 135.00[132.00,138.00] | 136.00[133.00,138.00] | 0.037 |
| Potassium |  |  |  |
| (moles/volume) | 4.20[4.00,4.60] | 4.10[3.80,4.40] | <0.001 |
| Chloride |  |  |  |
| (moles/volume) | 96.00[92.00,99.00] | 97.00[94.00,99.00] | 0.010 |
| Lactate |  |  |  |
| (moles/volume) | 1.60[1.20,2.30] | 1.40[1.10,1.80] | 0.003 |
| Bicarbonate |  |  |  |
| (moles/volume) | 23.00[21.00,25.00] | 24.00[22.00,26.00] | 0.010 |
| BUN |  |  |  |
| (mass/volume) | 20.00[14.00,33.00] | 13.00[9.00,18.00] | <0.001 |
| SCR |  |  |  |
| (mass/volume) | 1.23[0.85,1.62] | 0.88[0.69,1.06] | <0.001 |
| GFR |  |  |  |
| (ml/min) | 57.00[43.00,86.00] | 96.00[75.00,112.00] | <0.001 |
| Glucose |  |  |  |
| (mass/volume) | 130.00[110.00,181.00] | 119.00[106.00,142.00] | 0.005 |

Abbreviations: CAD: coronary artery disease; COPD: chronic obstructive pulmonary disease; OLD: other lung diseases including asthma; ACEI: angiotensin converting enzyme inhibitor; ARB: Angiotensin receptor blocker; NSAID: non-steroidal anti-inflammatory drug; T: temperature; SaO2: artery oxygen saturation; PR: respiration rate; HR: heart rate; SBP: systolic blood pressure; MAP: mean blood pressure; AST: aspartate aminotransferase; ALT: alanine aminotransferase; PCT: procalcitonin; CRP: C-reactive protein; BUN: blood urea nitrogen; SCR: serum creatinine; GFR: glomerular filtration rate.

Supplementary Table 2. The parameters of the final multifactor model for AKI

| **Factors** | **Estimate** | **SE** | **Z** | **P** | **Odds Ratio** | **95%CI** |
| --- | --- | --- | --- | --- | --- | --- |
| GFR | -0.033 | 0.005 | -6.300 | <0.001 | 0.968 | 0.958-0.978 |
| SaO2 | -0.090 | 0.021 | -4.195 | <0.001 | 0.914 | 0.875-0.953 |
| PCT | 0.668 | 0.308 | 2.166 | 0.030 | 1.950 | 1.078-3.662 |
| CRP | 0.033 | 0.018 | 1.827 | 0.068 | 1.034 | 0.997-1.071 |
| CAD | 0.553 | 0.365 | 1.515 | 0.130 | 1.738 | 0.840-3.527 |

Abbreviations: CAD: coronary artery disease; SaO2: artery oxygen saturation; PCT: procalcitonin; CRP: C-reactive protein; GFR: glomerular filtration rate.
